# Celecoxib ameliorates diabetic sarcopenia by inhibiting inflammation, stress response, mitochondrial dysfunction, and subsequent activation of the protein degradation systems

**DOI:** 10.3389/fphar.2024.1344276

**Published:** 2024-01-19

**Authors:** Chunyan Deng, Chunfeng Lu, Kexin Wang, Mengyuan Chang, Yuntian Shen, Xiaoming Yang, Hualin Sun, Xinlei Yao, Chunjian Qiu, Feng Xu

**Affiliations:** ^1^ Department of Endocrinology, Affiliated Hospital 2 of Nantong University and First People’s Hospital of Nantong City, Nantong, China; ^2^ Key Laboratory of Neuroregeneration of Jiangsu and Ministry of Education, Co-Innovation Center of Neuroregeneration, NMPA Key Laboratory for Research and Evaluation of Tissue Engineering Technology Products, Nantong University, Nantong, China; ^3^ Department of Endocrinology, Jinling Hospital, Nanjing University School of Medicine, Nanjing, China

**Keywords:** diabetic sarcopenia, celecoxib, inflammation, oxidative stress, protein degradation systems

## Abstract

**Aim:** Diabetic sarcopenia leads to disability and seriously affects the quality of life. Currently, there are no effective therapeutic strategies for diabetic sarcopenia. Our previous studies have shown that inflammation plays a critical role in skeletal muscle atrophy. Interestingly, the connection between chronic inflammation and diabetic complications has been revealed. However, the effects of non-steroidal anti-inflammatory drug celecoxib on diabetic sarcopenia remains unclear.

**Materials and Methods:** The streptozotocin (streptozotocin)-induced diabetic sarcopenia model was established. Rotarod test and grip strength test were used to assess skeletal muscle function. Hematoxylin and eosin and immunofluorescence staining were performed to evaluate inflammatory infiltration and the morphology of motor endplates in skeletal muscles. Succinate dehydrogenase (SDH) staining was used to determine the number of succinate dehydrogenase-positive muscle fibers. Dihydroethidium staining was performed to assess the levels of reactive oxygen species (ROS). Western blot was used to measure the levels of proteins involved in inflammation, oxidative stress, endoplasmic reticulum stress, ubiquitination, and autophagic-lysosomal pathway. Transmission electron microscopy was used to evaluate mitophagy.

**Results:** Celecoxib significantly ameliorated skeletal muscle atrophy, improving skeletal muscle function and preserving motor endplates in diabetic mice. Celecoxib also decreased infiltration of inflammatory cell, reduced the levels of IL-6 and TNF-α, and suppressed the activation of NF-κB, Stat3, and NLRP3 inflammasome pathways in diabetic skeletal muscles. Celecoxib decreased reactive oxygen species levels, downregulated the levels of Nox2 and Nox4, upregulated the levels of GPX1 and Nrf2, and further suppressed endoplasmic reticulum stress by inhibiting the activation of the Perk-EIF-2α-ATF4-Chop in diabetic skeletal muscles. Celecoxib also inhibited the levels of Foxo3a, Fbx32 and MuRF1 in the ubiquitin-proteasome system, as well as the levels of BNIP3, Beclin1, ATG7, and LC3Ⅱ in the autophagic-lysosomal system, and celecoxib protected mitochondria and promoted mitochondrial biogenesis by elevating the levels of SIRT1 and PGC1-α, increased the number of SDH-positive fibers in diabetic skeletal muscles.

**Conclusion:** Celecoxib improved diabetic sarcopenia by inhibiting inflammation, oxidative stress, endoplasmic reticulum stress, and protecting mitochondria, and subsequently suppressing proteolytic systems. Our study provides evidences for the molecular mechanism and treatment of diabetic sarcopenia, and broaden the way for the new use of celecoxib in diabetic sarcopenia.

## 1 Introduction

Diabetes mellitus (DM) is one of the most prevalent chronic metabolic diseases, characterized by high blood glucose levels (Shen et al., 2022), and DM is also one of the leading causes of death and disability worldwide (Collaborators, 2023). Prolonged exposure to high glucose environment can lead to multiple chronic complications including diabetic sarcopenia (Gispen and Biessels, 2000; Shen et al., 2022; Ding et al., 2023). Diabetic sarcopenia (or skeletal muscle atrophy caused by DM) a common clinical disease, and numerous clinical studies have reported the declines of mass, strength, and function in muscle of diabetic patients (Cohen et al., 2015; Guerrero et al., 2016; Chazaud and Mounier, 2021). Diabetic sarcopenia not only has adverse effects on the treatment of DM, but also significantly affects exercise function and quality of life, potentially increasing the risk of premature death in patients (Izzo et al., 2021). Currently, there are no effective therapeutic strategies for diabetic sarcopenia.

Skeletal muscle atrophy is caused by an imbalance between protein synthesis and degradation, leading to the loss of muscle mass (Qiu et al., 2018; Wan et al., 2020; Ma et al., 2021; Baehr et al., 2022). Inflammation, oxidative stress, and impaired mitochondrial function are considered important upstream signals in skeletal muscle atrophy caused by various diseases (Shen et al., 2019; Huang et al., 2020; Shen et al., 2020; Sun et al., 2021; Ji et al., 2022; Zhang H. et al., 2023; Chen et al., 2023). Subsequently, they activate two major downstream proteolytic systems: the ubiquitin-proteasome system and the autophagic-lysosomal system, ultimately resulting in the occurrence of skeletal muscle atrophy (Lee et al., 2020; Wang et al., 2021; Wang et al., 2022; Zhang et al., 2022). In addition, inflammation and oxidative stress caused by high blood glucose levels often result in peripheral nerve diseases (Eid et al., 2023). The motor endplates (MEPs) are important structures between motor neurons and skeletal muscle, which maintain muscle function through signal transmission between motor neurons and muscle cells (Yin et al., 2019). Diabetic mice had acceleration of loss of both neuromuscular junctions (NMJ) and MEPs innervation (Francis et al., 2011). Furthermore, endoplasmic reticulum (ER) is essential for insulin production and secretion, and ER stress has been associated with diabetes (Yao et al., 2023).

Inflammation plays a crucial role in the process of skeletal muscle atrophy (Ji et al., 2022). It is noteworthy that systemic inflammation contributes to diabetic sarcopenia via decreased muscle protein synthesis and increased ubiquitin-proteasome and lysosomal-proteasome mediated protein degradation in DM (Donath, 2014; Perry et al., 2016; Shen et al., 2022). The evidence suggests that the inflammation-sensitive nuclear factor kappa B (NF-κB) and signal transducer and activator of transcription 3 (STAT3) pathways may contribute to muscle atrophy in T2DM (Perry et al., 2016). In a high-glucose environment, the production of a large number of oxygen free radicals and advanced glycation end products (AGEs) promotes the occurrence of diabetic skeletal muscle inflammation (Purnamasari et al., 2022), which leads to the secretion of myokines that are detrimental to diabetic complications recovery (Forbes and Cooper, 2013). However, there are few reports linking diabetic sarcopenia and anti-inflammatory effects. The bone morphogenetic protein 7 (BMP-7) had been shown to inhibit inflammation-induced muscle atrophy and improve muscle dysfunction caused by streptozotocin (STZ)-induced diabetes, its mechanism of action remains unclear (Aluganti Narasimhulu and Singla, 2021). The low-dose aspirin, a nonsteroidal anti-inflammatory drug (NSAID), had been reported in selected cases of bilateral thigh diabetic muscle infarction with controlled blood glucose levels. However, its duration of efficacy is short and it was associated with greater risk of gastrointestinal problems (Horton et al., 2016; Bako et al., 2019; Liu et al., 2023). Thus, it is still necessary to develop novel safe and effective anti-inflammatory drugs to improve inflammation and thus improve diabetic muscle sarcopenia.

COX2 plays an important role in inflammation and has been signaled as a target for anti-inflammation. Celecoxib is the potent, selective COX2 inhibitor (Cruz et al., 2022), and it reduces the production of inflammatory prostaglandins to achieve anti-inflammatory effects (Cruz et al., 2022). Inhibition of the COX2 pathway has been shown to improve muscle quality (Trappe et al., 2016; Zhang et al., 2022). Celecoxib has also been proven to be effective in various clinical trials, including bladder cancer (Kelly et al., 2019), breast cancer (Giacchetti et al., 2017), colorectal adenomas (Bertagnolli et al., 2006), familial adenomatous polyposis (Lynch et al., 2016), and bipolar disorder intervention (Husain et al., 2020), as well as arthritis (Yeomans et al., 2018). Its anti-inflammatory effects have also been demonstrated to improve liver fibrosis (Gao et al., 2016) and tendon tissue fibrosis (Jiang et al., 2014). Our previous study had shown that celecoxib could effectively alleviate denervation-induced skeletal muscle atrophy (Zhang et al., 2022). However, its role in diabetic sarcopenia has not been reported.

Currently, STZ-induced diabetes is the best model for studying muscle atrophy (Furman, 2015). In this study, we aim to explore the effects of celecoxib, a NSAID, on STZ-induced diabetic sarcopenia. The inflammation, oxidative stress, ER stress, upregulated autophagic-lysosomal and ubiquitin-proteasome systems, and mitochondrial dysfunction may all contribute to the development of muscle atrophy in diabetic muscles. However, celecoxib could alleviate STZ-induced diabetic sarcopenia through inhibiting inflammation, oxidative stress, ER stress, autophagic-lysosomal system, ubiquitin-proteasome system, and mitochondrial dysregulation. Our study provides evidences for the molecular mechanism and treatment of diabetic sarcopenia, and broadens the way for the new use of celecoxib in diabetic sarcopenia.

## 2 Methods

### 2.1 Animals

Animal experiments were approved by the Jiangsu Provincial Laboratory Animal Management Committee (No. 20180305-004). All animal studies were conducted according to the AAALAC and the IACUC guidelines. Male ICR mice (25 g±2 g, 8–10 weeks) were provided by the Experimental Animal Center of Nantong University and housed in a temperature-controlled room at 22 C with a 12 h light/dark cycle, with free access to water and diets. The mice were divided into control group (Control) and streptozotocin (STZ)-induced diabetes group. The mice were fasted for 15 h overnight, and then were injected with STZ (#S0130, Sigma) at a single dose of 150 mg/kg (dissolved in 100 mmol/L citrate buffer, pH 4.5) in the diabetes group. The mice received an equal volume of citrate buffer in control group. Random blood glucose was measured on the third and seventh day, and if the levels of blood glucose were consistently above 16.7 mmol/L, the mice were considered diabetic mice and included in the study. Subsequently, the diabetic mice were randomly divided into two groups: the diabetes group (DM) and the diabetes group treated with celecoxib (at a dose of 5 mg/kg/d, Pfizer, NY, United States) (DM + CXB), given through intraperitoneal injection for 28 consecutive days. The mice in control and DM groups were given an equal volume of normal saline. The motor functions were used to initially assess the development of diabetic sarcopenia (diabetes-induced muscle atrophy). After 4 weeks of celecoxib treatment, the mice were euthanized with pentobarbital sodium injection, and muscle samples were immediately collected to further analysis. Subsequently, the samples were either frozen at −80°C for later experiments, fixed in 4% paraformaldehyde (PFA, #G1101, Servicebio), or embedded in pre-cooled OCT (#4583, Leica, Germany) for subsequent experiments.

### 2.2 Motor function test

(1) Rotarod Test

In the week before formal testing, each mouse was subjected to adaptive running training, with the speed gradually increasing from 5 r/min to 10 r/min to 15 r/min, for 5 min each time, three consecutive times. If the mouse was initially unable to run, tail assistance was provided. For formal testing, the mouse was placed on the lane of the rotarod apparatus (Panlab, LE8500) with the minimum speed at 4 r/min. After the mouse stabilized, the speed was gradually increased from 4 r/min to 40 r/min within 10 min. The time for the mouse to fall from the lane was recorded for three consecutive times, and the longest fall time was recorded.

(2) Hindlimb Grip Test

The grip strength meter (Shanghai HaoHao, SH-10) was placed flat on the cage cover. The tail of the mouse was held to control the mouse’s body parallel to the direction of force. The hind paw of the mouse clasped the hook of the grip strength meter. Simultaneously, force was applied to the tail of the mouse in the opposite direction of the grip strength meter, until the hind paw of the mouse released. The test was conducted six times at 1-min intervals, and the average muscle strength value was calculated.

### 2.3 Histological staining

The tibialis anterior fixed in 4% PFA was dehydrated by gradient alcohol and embedded in OCT. After he tibialis anterior was sectioned (transverse and longitudinal sections of 10 μm), they were dried at 37°C for 2 h and used for subsequent experiments. For hematoxylin-eosin (H&E) Staining: after washing with PBS to remove OCT, the sections were stained with hematoxylin and eosin, and images were captured using an optical microscope to observe inflammatory infiltration in tibialis anterior. For immunofluorescence staining: the sections were blocked by 5% BSA, then incubated with anti-laminin (#ab11575, Abcam, United Kingdom), anti-CD68 (#38005, Signalway Antibody, United States), anti-NF200 (#N2912, Sigma, United States) and anti-α-BTX (#MKCK3522, Sigma, United States). The secondary antibody was Alexa Fluor^®^488 (#ab150073, Abcam, United Kingdom) or Alexa Fluor^®^594 (ab150116, Abcam, United Kingdom). The sections were sealed after staining with DAPI (#P0131, Beyotime, China). Images were captured using an SP5 confocal microscope (Leica, Wetzlar, Germany).

### 2.4 SDH staining

The fresh tibialis anterior were quickly embedded in OCT, then sliced (10 μm). The entire process was performed as quickly as possible, and the sections were stained with SDH staining reagent (#G2000, Solarbio, China). Images were captured using Zeiss microscope (Germany), and the proportion of positive muscle fibers was calculated.

### 2.5 ROS measurement

The level of reactive oxygen species (ROS) in the tibialis anterior was determined using the DHE, a superoxide anion fluorescence probe (#S0063, Beyotime, China). The sections of fresh tibialis anterior (10 μm) were washed in PBS for 10 min, then incubated with DHE (10 μM) for 30 min at room temperature in the dark. The sections were washed three times with PBS for 5 min and sealed. All images were captured using a Zeiss microscope (Germany). The fluorescent intensity was analyzed using Image J software.

### 2.6 Ultrastructural analysis

The tibialis anterior was prepared as a 1*1*3 mm^3^ cubic specimen and immersed in 2.5% glutaraldehyde followed by re-fixation in 1% osmium tetroxide. The section underwent rapid dehydration and was embedded in polymeric epoxy resin. Finally, the section was stained with 1% uranyl acetate and 0.3% lead citrate. The electron microscope section was photographed using a transmission electron microscope (HT7700, Hitachi, Tokyo, Japan).

### 2.7 Western blotting

Muscle tissue was lysed in a tissue lysis buffer containing proteinase inhibitor and phosphatase inhibitor. After centrifugation, the supernatant was collected and the protein concentration was determined using the BCA Protein Assay Kit (#P0010, Beyotime). An appropriate amount of protein was loaded onto 8%, 10%, or 12% one-step PAGE gels (#E303-11, Vazyme) according to the instructions. The separated proteins were then transferred to PVDF membranes activated by methanol. After blocking with 5% skim milk, the membranes were incubated with primary antibodies overnight at 4°C, followed by incubation with secondary antibodies at room temperature for 2 h on a horizontal shaker. The membranes were then washed and developed. The antibodies included: β-Tubulin (#ab6046), Cox2 (#ab15191), IL-1β(#ab254360), Nox2(#ab129068), Nox4(#ab109225), GPX1(#ab22604), Nrf2(#ab137550), Fbx32(#ab168372), MHC(#ab91506), Beclin1(#ab207612), ATG7(#ab133528), PGC1α(#ab191838), IL-6 (#ab9731), Sirt1 (#ab110304), BNIP3 (#ab10433), Goat Anti-Rabbit IgG H&L (HRP) (#ab205718), Goat Anti-Mouse IgG H&L (HRP) (#ab205719) from Abcam; CD68 (#38005), MuRF1 (#38580) from SAB; CD86 (#26903-1-AP), PTGES2 (#10881-1-AP), TNF-α (#60291-1-lg) from Proteintech; p-NF-κB (#3033S), p-Stat3 (Tyr705, #9145S), NLRP3(#30835S), Caspase1 (E9R2D, #83383S), Perk (#3192S), EIF-2α (#9722S), p-EIF-2α (#9721S), ATF-4 (#11815S), Chop (#2895S), Foxo3a (#12829S) from Cell Signaling Technology; p-Perk (#PA5-40294) from Invitrogen; LC3Ⅱ (#M186-3) from MBL.

### 2.8 Statistical analysis

All data are presented as mean ± standard deviation (SD). Statistical analysis was performed using GraphPad Prism 8.0.2 software. One-way ANOVA and Tukey’s analysis were used to detect differences and statistical significances. A *p*-value <0.05 was considered statistically significant.

## 3 Results

### 3.1 Celecoxib improved motor function in STZ-induced diabetic mice

**FIGURE 1:**
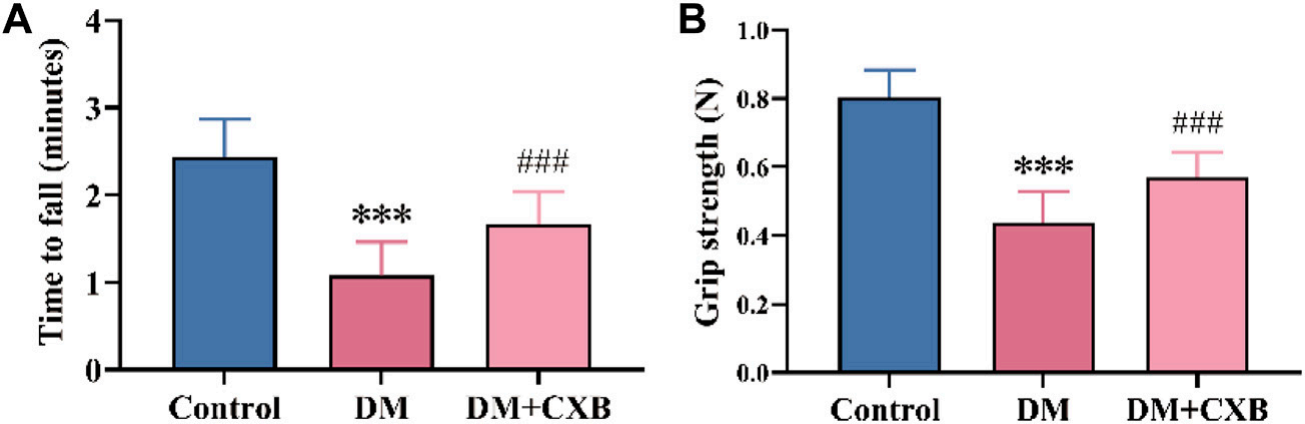
Celecoxib improved motor function in STZ-induced diabetic mice. **(A)** The times to fall of rotarod test (n ≥ 6); **(B)** Hindlimb grip strength (n ≥ 6). Data are presented as mean ± SD, ***, *p* < 0.001 DM *versus* control; ###, *p* < 0.001 DM + CXB *versus* DM. DM: diabetes mellitus; CXB: celecoxib.

After successful induction of diabetes using streptozotocin (STZ) in mice, we administered celecoxib treatment for 4 weeks. To evaluate the effects of celecoxib on motor function of diabetic mice, we measured the times to fall of rotarod test and hindlimb grip strength (Zhang et al., 2022). The results showed that compared to the control group, diabetic mice exhibited a significant decreased minute of rotarod test (Figure 1A) and a weakened hindlimb grip strength (Figure 1B), indicating impaired motor function and the possibility of diabetic sarcopenia (diabetes-induced skeletal muscle atrophy) in the muscle of diabetic mice. However, celecoxib showed a significant improvement in rotarod test and hindlimb grip strength in mice (Figures 1A,B). These results suggested that celecoxib significantly improves motor function of muscle in diabetic mice.

### 3.2 Celecoxib effectively alleviated diabetes-induced skeletal muscle atrophy

**FIGURE 2:**
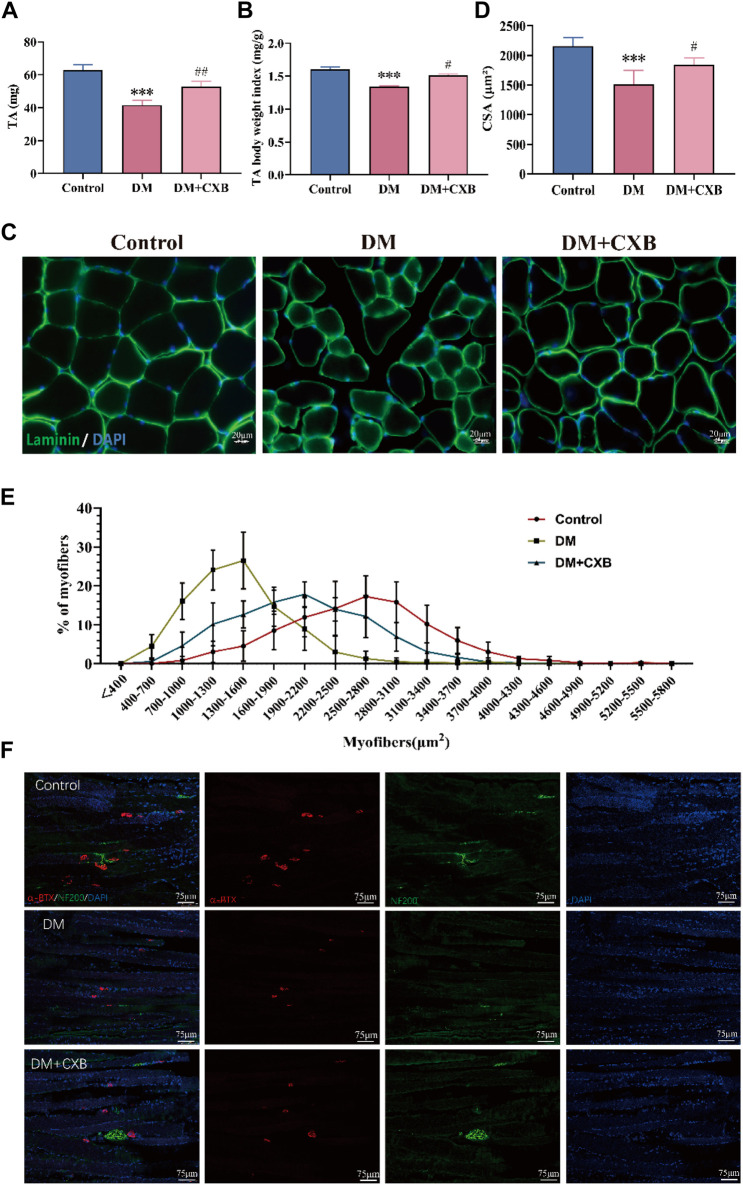
Celecoxib alleviated diabetes-induced skeletal muscle atrophy. **(A)** The weight of tibialis anterior (n ≥ 6); **(B)** The index of tibialis anterior weight/body weight (n ≥ 6); **(C)** Immunofluorescence staining of laminin in tibialis anterior, scale bar = 20 μm; **(D)** Cross-sectional area (CSA) of myofibers in tibialis anterior (n = 5); **(E)** Distribution of cross-sectional area of myofibers in tibialis anterior (n = 5); **(F)** Immunofluorescence staining of NF200 and α-BTX in sections of tibialis anterior, scale bar = 75 µm. Data are presented as mean ± SD, ***, *p* < 0.001 DM *versus* control; #, *p* < 0.01 DM + CXB *versus* DM; ##, *p* < 0.01 DM + CXB *versus* DM; ###, *p* < 0.001 DM + CXB *versus* DM. DM: diabetes mellitus; CXB: celecoxib.

We further examined the effects of celecoxib on diabetic sarcopenia. The results showed that compared to the control group, the muscle weight of the tibialis anterior (TA) and the index of TA weight/body weight were significantly reduced in DM group (Figures 2A, B). However, the reduces were restored in the diabetic mice treated with celecoxib (Figures 2A, B). Subsequently, we conducted laminin staining on TA muscle and observed a significant reduction in cross-sectional area (CSA) of myofibers in diabetic mice compared to the control group, indicating occurrence of evident atrophy in STZ-induced diabetic muscle (Figures 2C, D). However, the decrease in CSA of myofibers was inhibited by celecoxib (Figures 2C, D). The CSA distribution of myofibers in diabetic muscle was shifted to the left, which was inhibited by celecoxib (Figure 2E), consistent with the laminin staining. Moreover, under conditions of diabetes, fragmentation and a decrease in the quantity and size of the NMJs could be observed (Francis et al., 2011). To examine the restoration of diabetic neuropathy by celecoxib, we performed immunofluorescent staining with neurofilament 200 (NF200) to label nerve fibers and α-bungarotoxin (α-BTX) to label motor endplates in TA. The results showed that compared to the control group, the morphology and number of motor endplates were overall smaller and fewer in DM group, whereas they improved in the celecoxib group (Figure 2F). These indicated that NMJs were damaged during diabetic-induced muscle atrophy, and celecoxib had a certain protective effect in NMJs of skeletal muscles affected by diabetes. The above results demonstrated that diabetic sarcopenia was successfully constructed, and celecoxib effectively alleviated STZ-induced diabetic sarcopenia and protected the NMJs damaged by diabetes.

### 3.3 Celecoxib inhibited inflammation during diabetes-induced muscle atrophy

**FIGURE 3:**
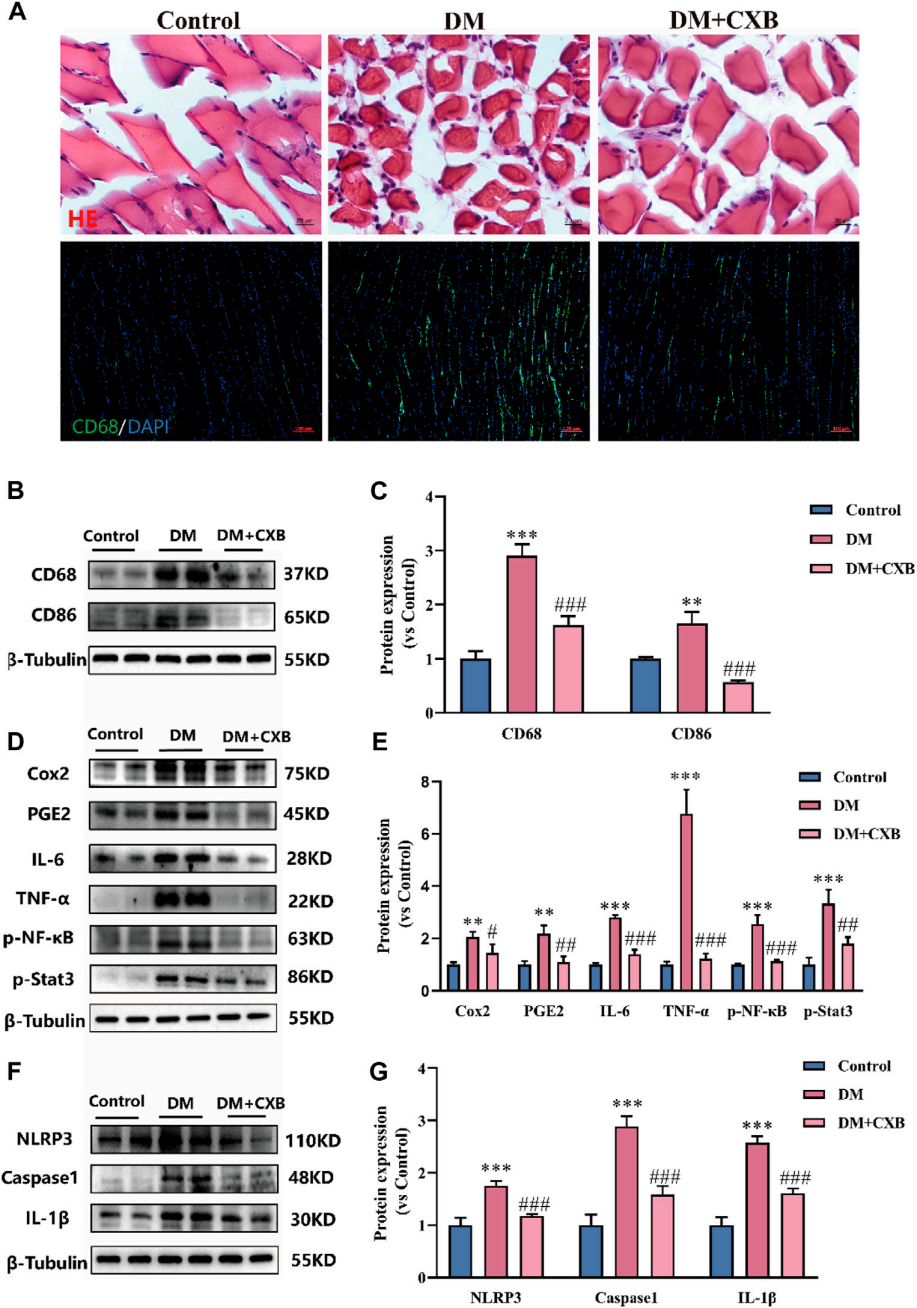
Celecoxib inhibited inflammation during diabetes-induced muscle atrophy. **(A)** Hematoxylin-eosin (H&E) staining of tibialis anterior, scale bar = 20 μm; and CD68 immunofluorescence staining of tibialis anterior, scale bar = 100 μm; **(B–C)** The levels of CD68 and CD86 were tested in tibialis anterior by Western blotting, and their quantitative analysis; **(D–E)** Western blotting analysis of the levels COX2, PGE2, IL-6, TNF-α, and the phosphorylation levels of NF-κB and Stat3 in tibialis anterior, and quantitative analysis; **(F–G)** The levels and grayscale value statistics of NLRP3, Caspase1, IL-1β of NLRP3 inflammasome pathway in tibialis anterior. Data are presented as mean ± SD, *n* ≥ 3; **, *p* < 0.01 DM versus control; ***, *p* < 0.001 DM versus control; #, *p* < 0.01 DM + CXB versus DM; ##, *p* < 0.01 DM + CXB versus DM; ###, *p* < 0.001 DM + CXB versus DM. DM: diabetes mellitus; CXB: celecoxib.

Celecoxib, an anti-inflammatory drug (Bertagnolli et al., 2006), may alleviate diabetic sarcopenia by exerting anti-inflammatory effects. We validated the anti-inflammatory effect of celecoxib during diabetes-induced muscle atrophy. The results showed that compared with control group, diabetes-induced atrophic muscle showed more infiltration of inflammatory cells, which were improved by celecoxib (Figure 3A). Macrophages are the main effector cells in inflammation (Ji et al., 2022), so we performed immunofluorescence staining for the marker CD68 of macrophage. The results showed that the levels of CD68 was significantly enhanced in diabetes-induced atrophic muscle, and it was reduced after celecoxib treatment (Figure 3A). Western blotting results of CD68 were consistent with immunofluorescence staining (Figure 3B). Moreover, compared with control group, the level of CD86, M1 macrophage marker, was significantly upregulated in atrophic muscle, and it was inhibited in the celecoxib group (Figures 3B–D). Celecoxib can inhibit the production of inflammatory PGE2 by inhibiting COX2, thus exerting anti-inflammatory effects (Cruz et al., 2022). Next, we measured the levels of COX2 and PGE2. The results showed that, compared with control group, the levels of COX2 and PGE2 were significantly upregulated in atrophic muscle, while celecoxib inhibited their increases (Figure 3E). The occurrence of diabetes-related inflammation is usually accompanied by the release of a large number of pro-inflammatory factors, which activate inflammatory signaling pathways (Shen et al., 2022). We verified that, compared with the control group, the levels of pro-inflammatory factors, IL-6 and TNF-α, and the phosphorylation levels of NF-κB and Stat3 were significantly increased in atrophic muscle, indicating activation of the inflammatory signaling pathways during diabetes-induced muscle atrophy. After celecoxib treatment, the levels of IL-6, TNF-α, p-NF-κB, and p-Stat3 were significantly reduced, indicating that the inflammation were significantly inhibited by celecoxib (Figures 3E–K). In addition, the NLRP3-mediated inflammasome pathway plays an important role in muscle atrophy (Zhang et al., 2022). The results showed that compared with the control group, the levels of NLRP3, Caspase1, and IL-1β were significantly upregulated in atrophic muscle, while celecoxib inhibited the increases in NLRP3-mediated inflammasome pathway (Figures 3L–O). These results indicated that celecoxib inhibited inflammation during diabetes-induced muscle atrophy by inhibiting the TNF-α/NF-κB, IL-6/Stat3, and NLRP3 inflammasome pathways.

### 3.4 Celecoxib alleviated oxidative stress during diabetes-induced muscle atrophy

**FIGURE 4:**
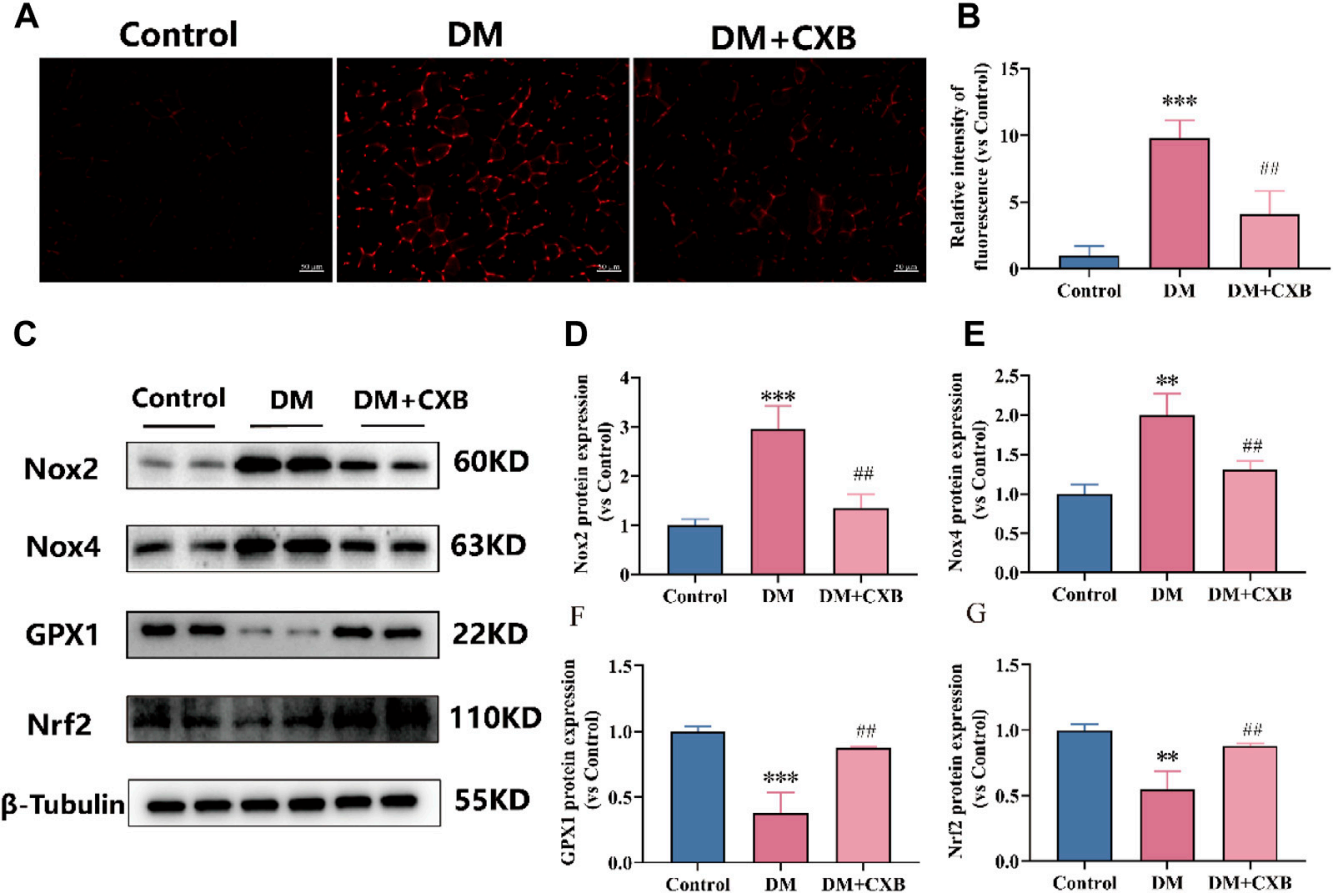
Celecoxib alleviated oxidative stress during diabetes-induced muscle atrophy. **(A)** Dihydroethidium (DHE) staining in tibialis anterior of mice; **(B)** Relative fluorescence intensity of DHE (n = 3), scale bar = 50 μm; **(C)** Western blotting results of NOX2, NOX4, GPX1 and Nrf2. **(D–G)** The statistics of NOX2, NOX4, GPX1 and Nrf2. Data are presented as mean ± SD, n ≥ 3; **, *p* < 0.01 DM *versus* control; ***, *p* < 0.001 DM *versus* control; ##, *p* < 0.01 DM + CXB *versus* DM. DM: diabetes mellitus; CXB: celecoxib.

Chronic inflammation in metabolic diseases can further increase the production of reactive oxygen species (ROS), leading to oxidative stress (Newsholme et al., 2016). To investigate whether celecoxib has antioxidant effects during diabetes-induced muscle atrophy, we first measured the levels of ROS in the tibialis anterior of mice using DHE probe. The results showed that the level of ROS was significantly increased in atrophic tibialis anterior, while it was significantly reduced after celecoxib treatment (Figures 4A, B). To understand how celecoxib affects the levels of ROS, we further performed western blotting to detect the levels of NADPH oxidases 2 (Nox2) and NADPH oxidases 4 (Nox4), which promote ROS production, as well as the levels of glutathione peroxidase 1 (GPX1) and antioxidant-related transcription factor Nrf2, which protect the body from oxidative stress (Zorov et al., 2014). The results showed that the levels of Nox2 and Nox4 were significantly increased, while GPX1 and Nrf2 were significantly downregulated in atrophic muscles. Celecoxib significantly reversed these phenomena (Figures 4C–G). The above results indicated that celecoxib alleviated oxidative stress during diabetes-induced muscle atrophy.

### 3.5 Celecoxib inhibited the excessive activation of endoplasmic reticulum stress during diabetes-induced muscle atrophy

**FIGURE 5:**
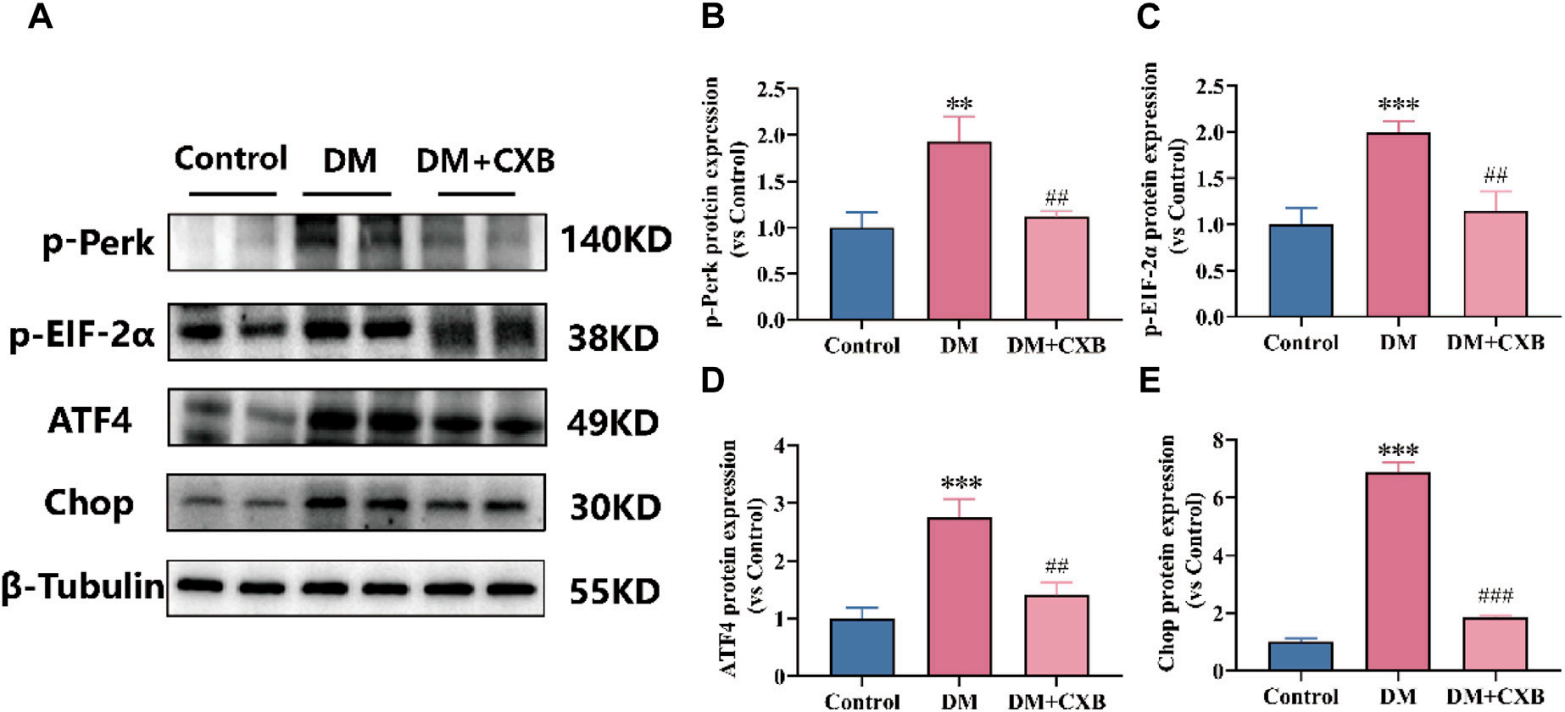
Celecoxib inhibited the excessive activation of ER stress during diabetes-induced muscle atrophy. **(A)** Western blotting results of p-Perk, p-EIF-2α, ATF4 and Chop. **(B–E)** The grayscale value statistics of p-Perk, p-EIF-2α, ATF4 and Chop. Data are presented as mean ± SD, n ≥ 3; **, *p* < 0.01 DM *versus* control; ***, *p* < 0.001 DM *versus* control; ##, *p* < 0.01 DM + CXB *versus* DM; ###, *p* < 0.001 DM + CXB *versus* DM. DM: diabetes mellitus; CXB: celecoxib.

Based on our above results, diabetes-induced atrophic muscles showed inflammation (Figure 3). The endoplasmic reticulum (ER) stress can trigger inflammatory pathways via the NF-κB pathway (Keestra-Gounder et al., 2016). To understand the effects of celecoxib on ER stress during diabetes-induced muscle atrophy, we used western blotting to detect the levels of p-Perk, p-EIF-2α, ATF4 and Chop, which related to ER stress. The results showed that the levels of p-PerK, p-EIF-2a, ATF4, and Chop were significantly upregulated in atrophic tibialis anterior, while celecoxib inhibited the upregulation of these (Figure 5). These results indicated that celecoxib inhibited the excessive activation of ER stress, possibly due to its anti-inflammation, thereby protecting against diabetes-induced muscle atrophy.

### 3.6 Celecoxib attenuated the activation of the ubiquitin-proteasome system during diabetes-induced muscle atrophy

**FIGURE 6:**
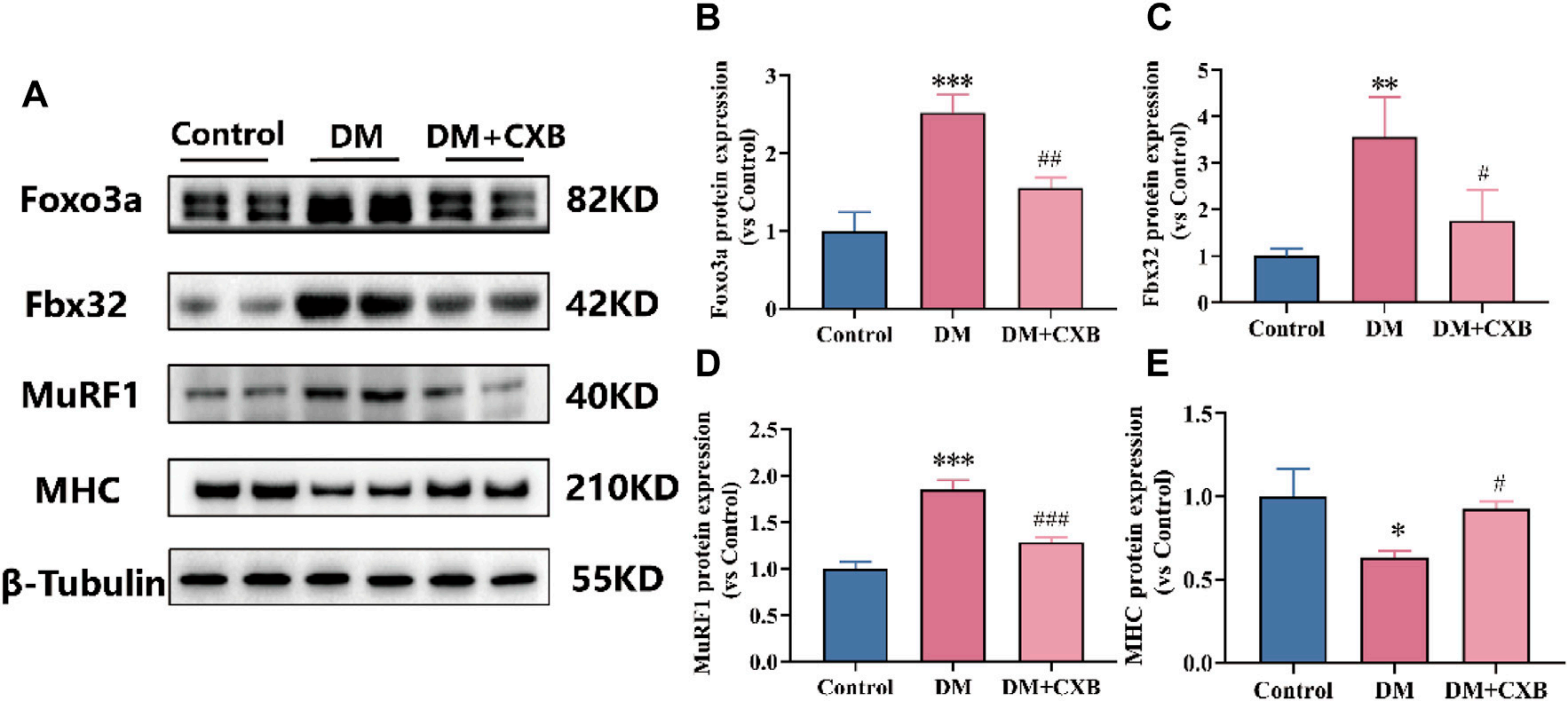
Celecoxib attenuated the activation of the ubiquitin-proteasome system during diabetes-induced muscle atrophy. **(A)** Western blotting results of Foxo3a, Fbx32, MuRF1 and MHC. **(B–E)** The grayscale value statistics of Foxo3a, Fbx32, MuRF1 and MHC. Data are presented as mean ± SD, n ≥ 3; *, *p* < 0.1 DM *versus* control; **, *p* < 0.01 DM *versus* control; ***, *p* < 0.001 DM *versus* control; #, *p* < 0.01 DM + CXB *versus* DM; ##, *p* < 0.01 DM + CXB *versus* DM; ###, *p* < 0.001 DM + CXB *versus* DM. DM: diabetes mellitus; CXB: celecoxib.

The inflammation can activate the ubiquitin-proteasome system by FoxO and downstream E3 ubiquitin ligases, and eventually leading to muscle atrophy in diabetes (Shen et al., 2022). To investigate the effect of celecoxib on the ubiquitin-proteasome system during diabetes-induced muscle atrophy, we identified the levels of Foxo3a, two E3 ubiquitin ligases (Fbx32 (also named Atrogin-1) and MuRF1) and myosin heavy chain (MHC) by western blotting. The results showed that compared with the control group, the levels of the Foxo3a, Fbx32 and MuRF1 were significantly upregulated in atrophic tibialis anterior, while celecoxib significantly reversed these phenomena (Figures 6A–D). Moreover, the levels of MHC were significantly downregulated in atrophic tibialis anterior, and celecoxib inhibited the downregulation (Figures 6A, E). These results indicated that celecoxib attenuated the activation of the ubiquitin-proteasome system during diabetes-induced muscle atrophy and has a protective effect on muscle.

### 3.7 Celecoxib attenuated the excessive activation of autophagy lysosome system during diabetes-induced muscle atrophy

**FIGURE 7:**
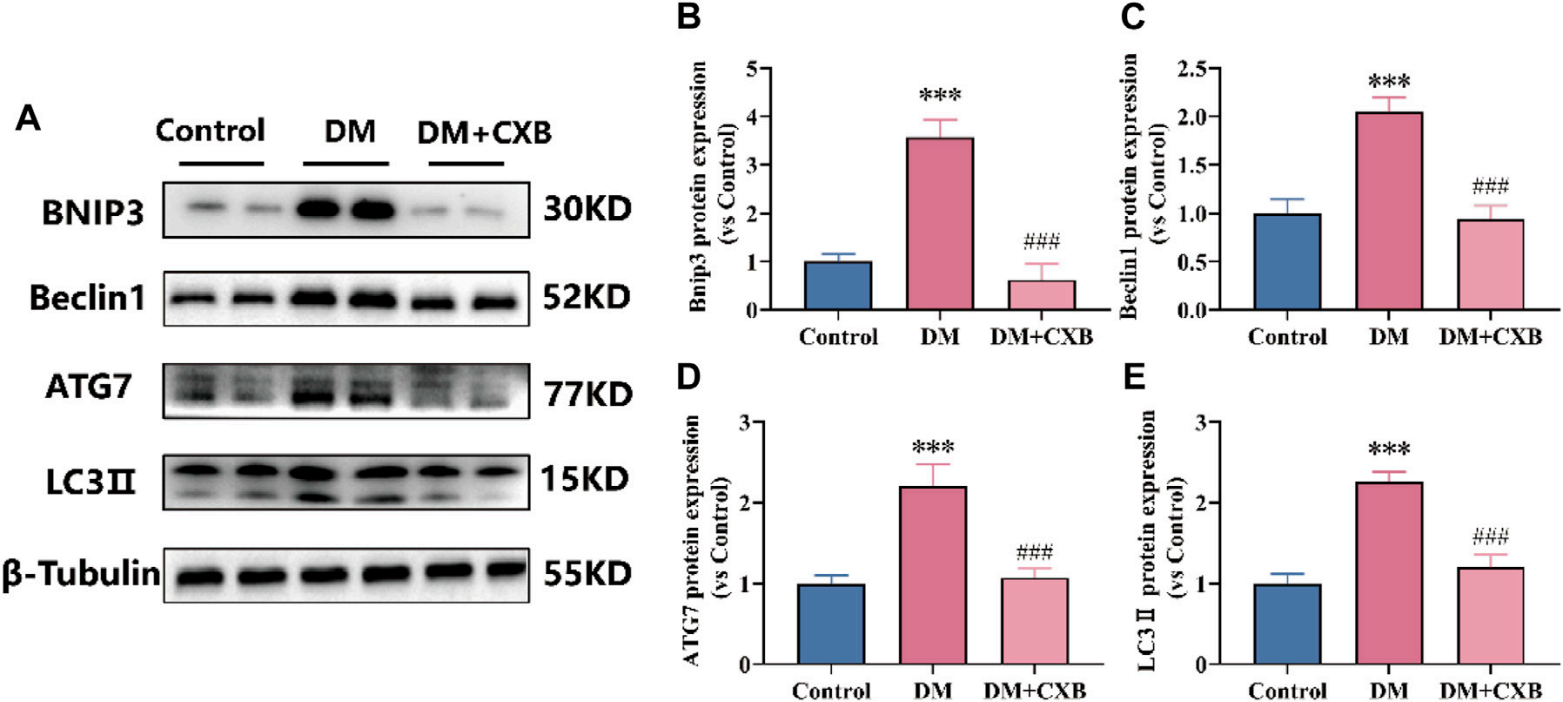
Celecoxib attenuated the excessive activation of autophagy lysosome system during diabetes-induced muscle atrophy. **(A)** Western blotting results of BNIP3, Beclin1, ATG7 and LC3-II. **(B–E)** The grayscale value statistics of BNIP3, Beclin1, ATG7 and LC3-II. Data are presented as mean ± SD, n ≥ 3; ***, *p* < 0.001 DM *versus* control; ###, *p* < 0.001 DM + CXB *versus* DM. DM: diabetes mellitus; CXB: celecoxib.

The autophagy lysosome system is also crucial for maintaining skeletal muscle homeostasis, but excessive activation of autophagy promotes muscle atrophy (Yin et al., 2021). FoxO3 regulates a number of essential autophagy genes, including Bcl2 family member BNIP3, LC3 II and ATG7 (Yin et al., 2021; Zhang et al., 2022). Beclin1, a component of VPS34 complex that regulates autophagy process, plays an important role in the autophagy lysosome system (Zhang et al., 2022). To determine the effects of celecoxib on autophagy lysosome system in diabetes-induced muscle atrophy, we examined the levels of BNIP3, Beclin1, LC3 II and ATG7 in tibialis anterior. Compared to control group, the levels of BNIP3, Beclin1, ATG7, and LC3 II were significantly upregulated in atrophic muscle, while celecoxib inhibited the upregulation of these autophagy-related proteins (Figures 7A–E). These results suggested that celecoxib can alleviate diabetes-induced muscle atrophy by attenuating the excessive activation of autophagy lysosome system.

### 3.8 Celecoxib protected mitochondrial morphology and function during diabetes-induced muscle atrophy

**FIGURE 8:**
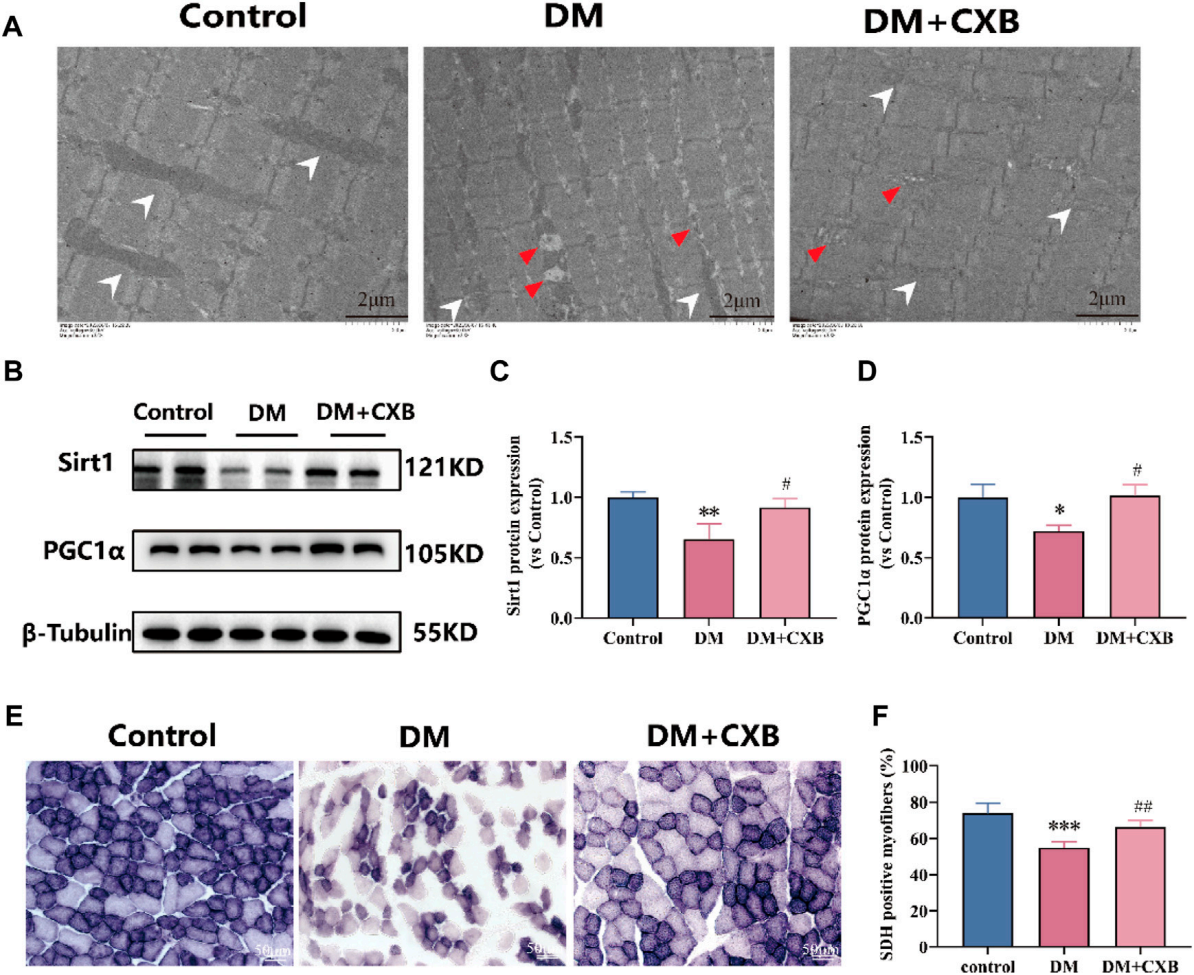
Celecoxib protected mitochondrial morphology and function during diabetes-induced muscle atrophy. **(A)** The electron microscopy image of mitochondria in tibialis anterior; Scale bar = 2 µm. **(B)** Western blotting results of SIRT1 and PGC1α in tibialis anterior; **(C–D)** The grayscale value statistics of SIRT1 and PGC1α, which maintain mitochondrial biogenesis; **(E)** The staining of succinate dehydrogenase (SDH) positive fibers; Scale bar = 50 µm. **(F)** The quantification of SDH positive fibers. Data are presented as mean ± SD, n ≥ 3; *, *p* < 0.1 DM *versus* control; **, *p* < 0.01 DM *versus* control; ***, *p* < 0.001 DM *versus* control; #, *p* < 0.01 DM + CXB *versus* DM; ##, *p* < 0.01 DM + CXB *versus* DM. DM: diabetes mellitus; CXB: celecoxib.

Diabetes affects the mass, strength, and function of skeletal muscles through disturbances in energy metabolism and mitochondrial dysfunction (Bianchi and Volpato, 2016). To evaluate the effects of celecoxib on mitochondria and metabolism during diabetes-induced muscle atrophy, we first observed the morphological changes of mitochondria using electron transmission microscopy. Compared to control group, the atrophic tibialis anterior showed disordered muscle filament arrangement and increased number of mitochondrial vacuoles, consistent with the autophagy results (Figure 7). However, celecoxib treatment group exhibited relatively orderly muscle filament arrangement and relatively intact mitochondria (Figure 8A). Furthermore, to understand the effects of celecoxib on mitochondrial biogenesis, we performed western blotting to detect the levels of Sirt1 and PGC1α. The results showed that compared to control group, the atrophic muscle exhibited significant downregulation of the levels of Sirt1 and PGC1α, while celecoxib significantly inhibited their downregulation (Figures 8B–D). Succinate dehydrogenase (SDH), one of the markers of mitochondrial function involved in the tricarboxylic acid cycle, provides energy for the body (Porporato et al., 2018). The results showed that compared to control group, the number of SDH-positive fibers had a significantly reduced in atrophic muscle, which was inhibited by celecoxib (Figures 8E, F). These results suggested that celecoxib could protect mitochondrial integrity, enhance mitochondrial biogenesis, maintain mitochondrial function during diabetes-induced muscle atrophy.

## 4 Discussion

Skeletal muscle accounts for approximately 40% of body weight and is the major metabolic organ of glucose and fatty acid. It is responsible for 80% of postprandial glucose metabolism stimulated by insulin and provides 70% of the body’s energy through mitochondrial respiration (Yap et al., 2020). In recent years, the number of individuals with diabetes has significantly increased. Diabetic muscle tissue is characterized by various metabolic abnormalities, which include insufficient levels of insulin, high levels of blood glucose, oxidative stress, and inflammation, which may lead to muscle dysfunction and muscle atrophy (Rohm et al., 2022). These can result in muscle weakness, exercise intolerance, and impaired quality of life and functional capacity (Regensteiner et al., 1995). The researchers have attempted to use various methods to treat diabetic sarcopenia (diabetes-induced skeletal muscle atrophy), such as resveratrol (Wang et al., 2018), exosomes derived from mesenchymal matrix cells (Song et al., 2023), SGLT2 inhibitors (Bamba et al., 2022), as well as a series of anti-inflammatory drugs such as IL-1 inhibitors, aspirin, and BMP-7 (Aluganti Narasimhulu and Singla, 2021; Ji et al., 2022). Despite promising results, the effectiveness of these treatments remains unclear, leading to their temporary suspension (Aluganti Narasimhulu and Singla, 2021; Ji et al., 2022). The mechanisms of diabetic sarcopenia are intricate and exercise remains the only effective intervention for it (Esefeld et al., 2021). Therefore, it is particularly important to develop novel preventive and curative strategies for diabetic sarcopenia.

Diabetes complications are chronic and systemic diseases, their treatments need to have long-term safety, which poses further challenges compared to drug development for acute and life-threatening diseases (Nauck et al., 2021). Thus, the development of drugs with clear targets for diabetes complications is particularly important. During diabetes-induced skeletal muscle atrophy, inflammation plays a critical role in causing protein imbalance and subsequent muscle atrophy (Ji et al., 2022; Shen et al., 2022). Celecoxib, a powerful anti-inflammatory drug with well-defined targets, has the potential for diabetic sarcopenia. In this study, the impact of celecoxib, an anti-inflammatory drug, on diabetic sarcopenia was investigated. We established a diabetic mouse model induced by STZ and regularly monitored blood glucose changes to understand whether the anti-inflammation of celecoxib could have a hypoglycemic effect. However, during the celecoxib treatment period, no significant changes were observed in blood glucose levels. Interestingly, we found that celecoxib had a positive impact on the weight, cross-sectional area, and exercise function of diabetes induced-atrophic muscles (Figures 1, 2), suggesting that although celecoxib did not have a hypoglycemic effect, it could alleviate skeletal muscle atrophy. In the future, the combination or sequential use of multiple anti-inflammatory drugs, or the combination of effective anti-inflammatory drugs with hypoglycemic drugs, could provide offer personalized treatments for patients with diabetes complications. Additionally, this also could provide understanding in treating diseases driven by inflammation.

Furthermore, one of the most common complications of diabetes is neuropathy (Rojas-Carranza et al., 2018). To validate the effects of celecoxib on diabetic neuropathy, we observed the morphology of neuromuscular junctions through staining, and found that diabetes induced-atrophic muscle showed smaller and fewer neuromuscular junctions, while celecoxib had a certain protect effect on them (Figure 2F). Our results suggested that diabetic neuropathy might be related to inflammation and celecoxib might alleviate neuropathy through its anti-inflammatory actions.

The inflammation caused by diabetes leads to muscle atrophy and muscle dysfunction (Aluganti Narasimhulu and Singla, 2021). Moreover, myokines and adipokines interact with each other to regulate metabolic homeostasis. Skeletal muscle atrophy also affects fat, leading to dysregulation of systemic homeostasis (Huang et al., 2022; Park and Choi, 2023). We further verified the presence of inflammation in atrophic skeletal muscles caused by diabetes (Figure 3). In particular, IL-6 is a double-edged sword, as it acts as both a myogenic factor, responsible for regulating muscle growth and regeneration and glucose homeostasis (Muñoz-Cánoves et al., 2013), and as well as a factor in insulin resistance, which works synergistically to cause inflammation and trigger β-cell apoptosis (Wei et al., 2008; Berchtold et al., 2016). In addition, the formation of NLRP3 inflammasomes triggers the upregulation of caspase-1 and IL-1β, leading to the formation of pyroptosis (Coll et al., 2022; Zhang Q. et al., 2023; He et al., 2023). Interestingly, the use of celecoxib alleviated the levels of inflammatory factors and the activation of proinflammatory pathways, thus paving the way for the alleviation of inflammation caused by diabetes and other diseases.

The hyperglycemic environment first causes high levels of ROS, leading to the occurrence of chronic inflammation (Newsholme et al., 2016). Celecoxib alleviated oxidative stress while exerting its anti-inflammatory effects, which confirms our previous research (Shen et al., 2022). It demonstrates the interplay between oxidative stress and inflammation, both of which contribute to the development of diabetes-induced skeletal muscle atrophy. Furthermore, endoplasmic reticulum (ER) stress is also activated during diabetes, and prolonged ER stress leads to cell damage, apoptosis, and tissue damage (Keestra-Gounder et al., 2016; He et al., 2023). It is noteworthy that celecoxib improved ER stress and mitigated the injury inflicted by ER stress on the diabetic muscles, possibly due to its anti-inflammatory properties. Inflammation, oxidative stress, and ER stress activate two major protein degradation pathways - the ubiquitin proteasome system and autophagy lysosome system, leading to increased protein degradation (Cohen et al., 2015; Ji et al., 2022). Moreover, mitophagy is a double-edged sword, excessive mitophagy would also promote cell death (Yang et al., 2022). In our study, we observed excessive upregulation of the levels of ubiquitin ligases and autophagy-related proteins, partly explaining the cause of diabetic sarcopenia, while celecoxib inhibited the excessive upregulation through its anti-inflammatory effects. In addition, mitochondrial biogenesis plays a key role in maintaining the dynamic balance of mitochondria to meet energy demands in muscle (Romanello and Sandri, 2015). Energy metabolism disorders and a shift from oxidative to glycolytic fiber types have been observed in diabetic muscles (Brandenburg et al., 1999; Oberbach et al., 2006). In our study, celecoxib increased the number of positive fibers for SDH and the levels of Sirt1 and PGC1α, which are involved in energy metabolism and mitochondrial biogenesis, possibly enhancing energy metabolism in muscle.

It is noteworthy that celecoxib is usually indicated for the symptomatic treatment of osteoarthritis, rheumatoid arthritis and ankylosing spondylitis in adults in the EU. Celecoxib is used for patients at the recommended dosages of 200 or 400 mg/day (Moore et al., 2005), usually lasts 24 weeks, and it was generally well tolerated, with mild to moderate upper GI complaints being the most common adverse events. At recommended dosages, the risks of increased thrombotic cardiovascular events, or renovascular, hepatic or hypersensitivity reactions would be small (McCormack, 2011). In our study, the diabetic mice treated with celecoxib at a lower and safe dose of 5 mg/kg/d for 4 weeks. In conclusion, celecoxib should be used at the lowest effective dosage for the shortest possible duration to minimize any risk.

In summary, our study demonstrated that celecoxib, a specific non-steroidal anti-inflammatory drug targeting Cox2, had potent effects on skeletal muscle atrophy in STZ-induced diabetic mice. Celecoxib inhibited oxidative stress, ER stress, and inflammation and attenuated subsequent ubiquitin-proteasome and autophagy lysosome systems, alleviating diabetic sarcopenia. Moreover, celecoxib protected mitochondria and neuromuscular junctions in atrophic muscles. Our study establishes the links between hyperglycemia, inflammation, and muscle atrophy, which are crucial for preventing diabetic sarcopenia and laying foundations for improving physical function, quality of life, and late-life health in patients with diabetes.

## Data Availability

The raw data supporting the conclusion of this article will be made available by the authors, without undue reservation.
